# Prevalence of Abnormal Glucose Regulation according to Different Diagnostic Criteria in Ischaemic Stroke without a History of Diabetes

**DOI:** 10.1155/2018/8358724

**Published:** 2018-05-22

**Authors:** Xinmiao Zhang, Qiuyan Shi, Huaguang Zheng, Qian Jia, Xingquan Zhao, Liping Liu, Chunxue Wang, Xia Meng, Jing Jing, Yuesong Pan, Yilong Wang, Yongjun Wang

**Affiliations:** ^1^Department of Neurology, Beijing Tiantan Hospital, Capital Medical University, Beijing 100050, China; ^2^China National Clinical Research Center for Neurological Diseases, Beijing 100050, China; ^3^Center of Stroke, Beijing Institute for Brain Disorders, Beijing 100050, China; ^4^Beijing Key Laboratory of Translational Medicine for Cerebrovascular Disease, Beijing 100050, China; ^5^Department of Neurology, Affiliated Hospital, North China University of Science and Technology, Tangshan 063000, China

## Abstract

We aimed to investigate the prevalence and distribution of abnormal glucose regulation, including prediabetes and newly diagnosed diabetes, according to different criteria in ischaemic stroke patients without a history of diabetes. Data were derived from a representative cohort across China. Prediabetes was defined as fasting plasma glucose (FPG) 5.6–6.9 mmol/L or 2-hour oral glucose tolerance test (OGTT) 7.8–11.0 mmol/L or haemoglobin A1c (HbA1c) 5.7–6.4%. Newly diagnosed diabetes was defined as FPG ≥ 7.0 mmol/L, 2 h OGTT ≥ 11.1 mmol/L or HbA1c ≥ 6.5%. Among 1251 ischaemic stroke patients, 471 (37.5%) were detected as prediabetes and 539 (43.1%) were detected as newly diagnosed diabetes. Prediabetes was present in 118 (9.4%), 290 (23.2%) and 314 (25.1%) stroke patients, and newly diagnosed diabetes was present in 138 (11.0%), 370 (29.6%), and 365 (29.2%) stroke patients, based on FPG, 2 h OGTT, and HbA1c criteria, respectively. Dependency on FPG alone would have missed 74.9% of patients in the prediabetes range and 74.4% of patients in the diabetes range. Our study demonstrated a high prevalence of prediabetes and diabetes in ischaemic stroke patients without a history of diabetes. OGTT and HbA1c helped detect the majority of prediabetes and newly diagnosed diabetes in ischaemic stroke patients.

## 1. Introduction

Diabetes is one of the most severe chronic diseases, which is also a contributor to the development of ischaemic stroke [1–3]. The prevalence of diabetes has increased rapidly in recent decades, especially in Asia, which was reported to be approximately 11.6% [4, 5]. Prediabetes, as an intermediate metabolic state between normal glucose regulation and diabetes, has an increasing incidence and high risk of developing diabetes [6]. Abnormal glucose regulation, including prediabetes and diabetes, often develops and interacts with ischaemic stroke. Abnormal glucose regulation doubles the risk of ischaemic stroke and worsens survival of patients with acute stroke [3, 7–9]. Previous studies showed that about one-third of ischaemic stroke patients had prediabetes, and 17.5% to 37.5% of stroke patients without a history of diabetes had unrecognized newly diagnosed diabetes [10–16]. In addition, abnormal glucose regulation has been proved to be associated with poor prognosis for ischaemic patients and has been widely used to predict the prognosis after an acute stroke as an important risk factor [17–22]. Hence, abnormal glucose regulation of stroke patients might be a therapeutic target for secondary prevention. Therefore, accurate diagnoses of abnormal glucose regulation is of great importance.

In previous studies, abnormal glucose regulation was diagnosed mostly based on fasting plasma glucose (FPG) or random blood sugar on admission [10–22], a small proportion of which was diagnosed according to postload glucose (2 h oral glucose tolerance test [OGTT]) [14, 22–25]. Recently, haemoglobin A1c (HbA1c) criteria have also been suggested as a diagnostic supplement by the American Diabetes Association and the World Health Organization [26, 27]. As reported, glucose-based and HbA1c-based criteria were frequently discordant regarding abnormal glucose regulation diagnosis [28]. Thus, using three diagnostic criteria combined (FPG, 2 h OGTT, and HbA1c) could increase the prevalence of abnormal glucose regulation, which may lead to a more standard secondary prevention and more favourable outcome. However, few studies, except one small European cohort [29], have investigated the prevalence and detailed distribution of prediabetes and newly diagnosed diabetes according to these three different diagnostic criteria in ischaemic stroke patients without a history of diabetes.

In our study, we used the Abnormal Glucose Regulation in Patients with Acute Stroke across China (ACROSS-China) registry to examine the prevalence and detailed distribution of prediabetes and newly diagnosed diabetes in ischaemic stroke patients without a history of diabetes according to different screening criteria (FPG, 2 h OGTT, and HbA1c).

## 2. Material and Methods

### 2.1. Study Participants

Ischaemic stroke patients without a history of diabetes in the survey on ACROSS-China were included in our study, details on the design and major results of which have been published previously [30]. Briefly, ACROSS-China is a well-designed, nationwide prospective cohort study that aimed to investigate the prevalence and distribution of abnormal glucose regulation in hospitalized patients (aged ≥ 18 years) between 2008 and 2009 across China. ACROSS-China has excluded all patients receiving intravenous alimentation because they could not finish the OGTT test [22].

Acute ischaemic stroke was diagnosed according to the World Health Organization criteria [31] combined with brain computed tomography or magnetic resonance confirmation. Patients were diagnosed as acute ischaemic stroke when the following conditions were met: acute occurrence within 14 days of neurological deficit, focal or overall involvement of the nervous system; symptoms that lasted for >24 hours; and the exclusion of nonvascular causes (e.g., primary and metastatic neoplasms, postseizure paralysis, or head trauma) that led to brain function deficit and of intracerebral hemorrhage by computed tomography or magnetic resonance imaging. The definition of a history of diabetes was either a self-reported physician diagnosis of diabetes mellitus or hypoglycemic medications (e.g., insulin or sulfonylureas) before hospitalization [22]. The diagnostic criteria were consistent across all participating hospitals. Patients with insulin or oral hypoglycemic agents during hospitalization and with extremely high plasma glucose levels during hospitalization were excluded for the sake of patients' safety and lack of the OGTT results.

### 2.2. Data Collection

Patient baseline information was recorded at admission, including sex, age, height, weight, blood pressure, fasting glucose level, and triglyceride. Assessment of baseline vascular risk factors included history of stroke, hypertension, dyslipedaemia, atrial fibrillation, coronary heart disease, diabetes, and body mass index (BMI). The National Institutes of Health Stroke Scale (NIHSS) and Glasgow Coma Scale were used to evaluate the severity of neurologic impairment within 24 hours after admission and at discharge. The clinical subtypes of ischaemic stroke were classified according to the Oxfordshire Community Stroke Project criteria [32].

### 2.3. Assessment of Abnormal Glucose Regulation

Prediabetes was defined as FPG level of 5.6–6.9 mmol/L, 2 h OGTT level of 7.8–11.0 mmol/L, or HbA1c level of 5.7–6.4%. Newly diagnosed diabetes was defined as FPG ≥ 7.0 mmol/L, 2 h OGTT ≥ 11.1 mmol/L, or HbA1c ≥ 6.5%.

FPG, 2 h OGTT, and HbA1c levels were assessed in the study patients. A standard FPG and OGTT was performed in the ischaemic patients without previous diabetes during the morning hours (range: 07:00–11:00) on day 14 ± 3 after stroke onset or before discharge (if hospital stay < 14 days) according to the World Health Organization criteria [33]. After overnight fasting (at least 8 hours), the patients drank 250 mL of a solution that included 75 g of glucose within 3 minutes. Immediately before administration of the drink and after 2 hours, venous blood samples were collected in sodium fluoride tubes to measure plasma glucose. Fasting and 2 h OGTT plasma glucose levels were measured (mmol/L) via an automated glucose oxidation method. The first overnight fasting venous blood samples were drawn to measure the blood HbA1c levels following admission using high-performance liquid chromatographic analysis, which were aligned to the Diabetes Control and Complications Trial and the National Glycohemoglobin Standardization Program standards [34].

### 2.4. Statistical Analysis

We presented continuous variables as mean ± SD or median with interquartile range and categorical variables as percentages. The demographic and clinical characteristics of the stroke patients with prediabetes and newly diagnosed diabetes were compared with patients with normal glucose regulation using the *χ*^2^ test for categorical variables and *t*-test or Wilcoxon rank-sum test for continuous variables, respectively. We also evaluated the prevalence of patients with prediabetes and newly diagnosed diabetes based on FPG, 2 h OGTT, and HbA1c criteria. The correlations between any two of the three diagnostic criteria were estimated. The data were analysed with SAS version 9.4 statistical software (SAS Institute Inc., Cary, NC, USA). All reported *P* values were two-sided with *P* < 0.05 being considered significant.

### 2.5. Ethical Approval

The study was approved by the Ethics Committee of Beijing Tiantan Hospital and all participating hospitals, in compliance with the Declaration of Helsinki. Written informed consent was obtained from all participants. All the experiments described were performed in accordance with the approved guidelines.

## 3. Results

### 3.1. Study Participants and Characteristics

A total of 1251 ischaemic stroke patients without a history of diabetes were included in the final analysis. The patient selection has been previously reported [30]. The clinical characteristics of the included 1251 patients were shown in Tables 1 and 2. The average age was 62.2 ± 12.6 years and 789 (63.3%) patients were men. For patients with prediabetes, they were older and more likely to have an increased level of LDL at admission, an increased frequency of a history of hyperlipidaemia and atrial fibrillation, and an increased possibility of being large arterial atherosclerosis, developing pulmonary infection, and receiving statin and antihypertensive drugs, compared with normal glucose regulation patients. For patients with newly diagnosed diabetes, they were older and less likely to be men. In addition, they were more likely to have increased levels of triglyceride and LDL at admission, an increased frequency of a history of hypertension, and an increased possibility of being large arterial atherosclerosis, developing pulmonary infection, and receiving oral hypoglycaemic drugs, insulin, and antihypertensive drugs, compared with normal glucose regulation patients.

### 3.2. Prevalence and Distribution of Abnormal Glucose Regulation

**Figure 1:**
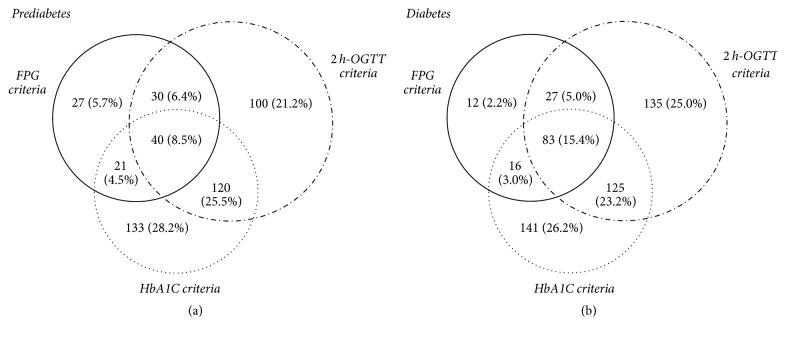
The prevalence and distribution according to different diagnostic criteria of prediabetes (a) and newly diagnosed diabetes (b) in ischaemic stroke patients without a history of diabetes. FPG criteria, fasting plasma glucose ≥ 7.0 mmol/L; 2 h OGTT criteria, 2-hour oral glucose tolerance test ≥ 11.1 mmol/L; HbA1c criteria, haemoglobin A1c ≥ 6.5%.

The prevalence and detailed distribution of prediabetes and newly diagnosed diabetes identified by the three different diagnostic criteria were shown in Figure 1. 471 stroke patients (37.5%) with prediabetes and 539 stroke patients (43.1%) with newly diagnosed diabetes were detected based on all three detecting methods combined (FPG, 2 h OGTT, and HbA1c). Prediabetes was present in 118 (9.4%), 290 (23.2%), and 314 (25.1%) of enrolled ischaemic stroke patients, based on FPG, 2-hour OGTT, and HbA1c criteria, respectively. Newly diagnosed diabetes was present in 138 (11.0%), 370 (29.6%), and 365 (29.2%) of enrolled stroke patients, based on FPG, 2 h OGTT, and HbA1c criteria, respectively. Prediabetes and newly diagnosed diabetes were identified more frequently using 2 h OGTT (23.2%, 29.6%) and HbA1c (25.1%, 29.2%) than by FPG (9.4%, 11.0%), respectively.

As shown in Figure 1, for patients with newly diagnosed diabetes, we detected 12 (2.2%) with isolated elevated FPG, 135 (25.0%) with isolated elevated 2 h OGTT, and 141 (26.2%) with isolated elevated HbA1c. The rest of the patients with newly diagnosed diabetes (46.6%) were diagnosed by the combined criteria. Using the traditional glucose-based criteria (FPG ≥ 7.0 mmol/L, or OGTT ≥ 11.1 mmol/L), 398 (31.8%) patients were diagnosed newly diabetes, and the total percentage increased to 43.1% with the supplement of the HbA1c criteria (HbA1c ≥ 6.5%). These distribution characteristics of the three different diagnostic criteria were also seen in the prediabetes patients. Dependency on FPG alone would have missed 74.9% (353/471) of patients in the prediabetes range and 74.4% (401/539) of patients in the diabetes range.

**Figure 2:**
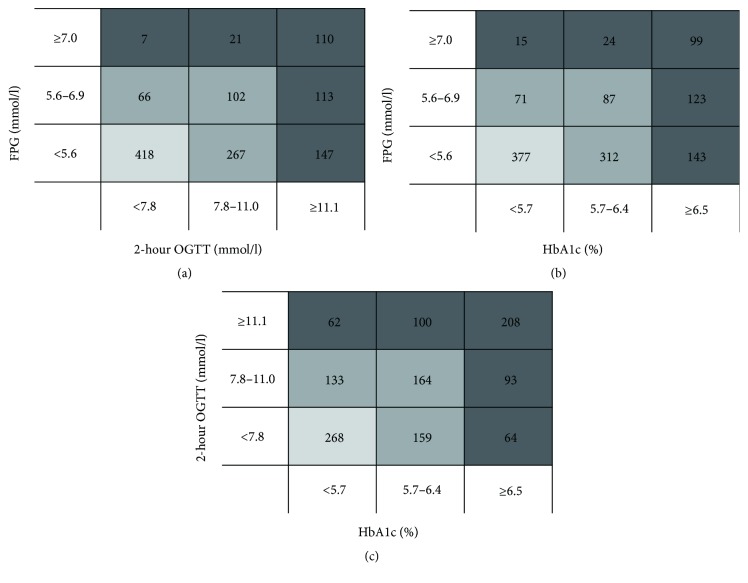
The figures (a–c) showed the correlative distribution between the different diagnostic methods. FPG, fasting plasma glucose; OGTT, oral glucose tolerance test; HbA1c, haemoglobin A1c.

The correlations between any two of the three diagnostic criteria were shown in Figure 2. Of the 832 patients with normal FPG level, 267 (32.1%) had impaired glucose tolerance and 312 (37.5%) had HbA1c levels in the prediabetes range. In addition, 147 (17.7%) patients had 2 h OGTT levels in the diabetes range, and 143 (17.2%) patients had HbA1c levels in the diabetes range. Furthermore, of the 281 patients with impaired fasting glucose, 113 (40.2%) and 123 (43.8%) had 2 h OGTT and HbA1c levels in the diabetes range, respectively.

## 4. Discussion

We investigated the prevalence and detailed distribution of prediabetes and newly diagnosed diabetes according to three different diagnostic criteria in ischaemic stroke patients without a history of diabetes. Our study demonstrated a high prevalence of prediabetes and newly diagnosed diabetes in ischaemic stroke patients. OGTT and HbA1c criteria helped detect the majority of prediabetes and unrecognized newly diabetes in ischaemic stroke patients.

Previous studies have demonstrated that the prevalence of prediabetes occurred in 31% to 38.5% of stroke patients and the prevalence of newly diagnosed diabetes in stroke patients was between 17.5 and 37.5% [10–16]. In our study, we found that prediabetes made up 37.6% of all ischaemic stroke patients without a history of diabetes based on three diagnosed criteria combined, as previous study reported. The newly diagnosed diabetes made up 43.1% of all ischaemic stroke patients without a history of diabetes, which was slightly more than that reported in previous study [29]. We infer that the most important reason was a high prevalence and low awareness of diabetes in China [4, 5]. A recent nationwide study showed that the prevalence of diabetes among Chinese aged 60 to 69 years was 25%, and the proportion of those knowing of their diabetes was only 40% [35].

The glucose-based (FPG and 2 h OGTT) and HbA1c-based criteria were frequently discordant regarding abnormal glucose regulation diagnosis [36]. Therefore, combining all three diagnostic criteria (FPG, 2 h OGTT, and HbA1c) may help detect more prediabetes and unrecognized newly diagnosed diabetes patients. Few studies, except for a European study [29], have investigated the prevalence and detailed distribution of prediabetes and newly diabetes diagnosed with these three criteria methods in ischaemic stroke patients. In this European study, however, the sample size was limited (*n* = 374) and the patients were confined to European stroke patients. In our study, prediabetes was present in 118 (9.4%) of the ischaemic stroke patients using the FPG criteria alone. By adding the 2 h OGTT criteria, the percentage increased to 27.0%. After adding the HbA1c criteria, the corresponding percentage reached 37.6%. For patients with newly diagnosed diabetes, the corresponding proportions based on the different criteria were 11.0%, 31.8%, and 43.1%, respectively. Therefore, using the combined diagnostic criteria, there were many more abnormal glucose regulation patients detected after adding patients with single high OGTT and HbA1c levels.

The OGTT and HbA1c criteria played an indispensable role in accurately diagnosing abnormal glucose regulation in our study. The majority of ischaemic stroke patients with prediabetes (94.3%) and newly diagnosed diabetes (97.8%) were detected based on 2 h OGTT or HbA1c criteria. Firstly, it was common for Asian population to be diagnosed prediabetes and diabetes by 2 h OGTT which considered normal according to FPG [37]. Previous studies [14, 23] have used OGTT to detect prediabetes and newly diagnosed diabetes in stroke patients and found a high prevalence. In our study, we also observed similar results in diagnosing prediabetes (61.6%, 290/471) and newly diagnosed diabetes (68.6%, 370/539) based on the 2 h OGTT criteria. Dependency on FPG levels alone would have missed 49.8% (414/832) of patients with an abnormal 2 h OGTT. In addition, OGTT test has been proved to be a better predictor of vascular events and poor prognosis than FPG levels [24, 25]. All of these studies indicate the importance of performing OGTT to detect abnormal glucose regulation among stroke patients without a previous history of diabetes. Secondly, HbA1c, which has recently been proposed as an alternative to diagnose diabetes [26], has helped avoiding omitting plenty undiagnosed prediabetes and diabetes patients. In our study, prediabetes was present in 25.1% (314/1251) and newly diagnosed diabetes was present in 29.1% (365/1251) of ischaemic stroke patients based on HbA1c levels, which identified twice as many as the FPG criteria alone. In addition, high HbA1c levels may illustrate persistent hyperglycaemia, which has been proved to be associated with worse functional outcomes [38, 39]. Furthermore, HbA1c levels can be used in stroke patients with dysphagia, which adds to the importance of HbA1c in stroke patients.

The early recognition of abnormal glucose regulation in ischaemic stroke patients is significant, as vascular complications are preventable through strict glycaemic control [40, 41]. As the indispensable components to diagnose prediabetes and newly diagnosed diabetes, OGTT and HbA1c should be attached importance equally as FPG did. Using single indicator (FPG, 2 h OGTT, or HbA1c) as the diagnostic criteria of prediabetes and newly diagnosed diabetes may lead to a large proportion of missed diagnoses. Overlooking OGTT and HbA1c examination may yield an important fraction of patients with abnormal glucose regulation. Therefore, our findings recommend the significance of integrated glucose examination at admission of stroke patients without a history of diabetes, which includes FPG, OGTT, and HbA1c, especially in the Chinese population.

Our study has several limitations. First, some ischaemic stroke patients were excluded because of the lack of OGTT (including dysphagia) and HbA1c data in the hospital, which may have led to bias. However, no significant differences in sex, vascular risk factors, or medical history were identified between the included and excluded patients. Second, all FPG and OGTT were tested on days 14 ± 3 after the stroke onset or before discharge (if hospital stay was <14 days) according to previous studies. However, it is difficult to avoid mixed patients with stress hyperglycemia, which could lead to bias. Third, the OGTT and HbA1c tests were not repeated according to the advice of the American Diabetes Association and newly diagnosed diabetes may be overestimated in our study. Finally, patients treated with insulin or oral hypoglycemic agents probably had diabetes even though FPG or 2-hour OGTT levels were within the normal range. In these patients, relationship between FPG or 2-hour OGTT and HbA1c could lead to a bias by the treatments.

## 5. Conclusion

Our findings demonstrated a high prevalence of prediabetes and newly diagnosed diabetes in ischaemic stroke patients without a history of diabetes. 2 h OGTT and HbA1c criteria helped detect the majority of prediabetes and unrecognized newly diabetes in ischaemic stroke patients.

## Figures and Tables

**Table 1:** Baseline characteristics of patients in different states of glucose regulation.

Variables	Normal glucose regulation (*n* = 241)	Prediabetes^*∗*^ (*n* = 471)	Newly diagnosed diabetes^‡^ (*n* = 539)	*P* value
Sex (men), *n* (%)	171 (71.0)	304 (64.8)	314 (58.6)	0.003
Age, y, mean ± SD	58.3 ± 12.6	61.8 ± 12.8	64.2 ± 11.9	<0.001
NIHSS score at admission, median (IQR)	4 (2–7)	4 (2–8)	4 (2–8)	0.93
Body mass index, kg/m2, mean ± SD	24.6 ± 3.5	24.7 ± 3.9	25.1 ± 3.8	0.07
Triglyceride at admission, mmol/L, mean ± SD	1.6 ± 1.1	1.7 ± 1.0	1.9 ± 1.2	<0.001
LDL at admission, mmol/L, mean ± SD	2.9 ± 0.9	3.1 ± 1.6	3.2 ± 1.5	0.02
HDL at admission, mmol/L, mean ± SD	1.2 ± 0.3	1.2 ± 0.3	1.2 ± 0.4	0.20
History of hypertension, *n* (%)	133 (55.2)	274 (58.2)	339 (62.9)	0.09
History of hyperlipidaemia, *n* (%)	18 (7.5)	68 (14.4)	63 (11.7)	0.02
History of atrial fibrillation, *n* (%)	9 (3.7)	37 (7.9)	25 (4.6)	0.03
History of coronary heart disease, *n* (%)	23 (9.5)	61 (13.0)	70 (13.0)	0.35
Smoking, *n* (%)				0.58
Current smoker	90 (37.3)	160 (34.0)	173 (32.1)	
Ever smoker	19 (7.9)	49 (10.4)	54 (10.0)	
Nonsmoker	132 (54.8)	262 (55.6)	312 (57.9)	
Medicine use during hospitalization, *n* (%)				
Oral hypoglycaemic drugs	1 (0.6)	7 (2.1)	59 (14.3)	<0.001
Insulin	2 (0.8)	8 (1.7)	67 (12.4)	<0.001
Antihypertensive drugs	0 (0.0)	11 (2.3)	175 (32.5)	<0.001
Diuretics	5 (2.1)	13 (2.8)	11 (2.0)	0.72
B-blockers	6 (2.5)	21 (4.5)	21 (3.9)	0.43
Statin	107 (44.4)	253 (53.7)	277 (51.4)	0.06
Antiplatelet	155 (64.3)	304 (64.5)	332 (61.6)	0.58
Anticoagulation	12 (5.0)	27 (5.7)	36 (6.7)	0.62
Pulmonary infection, *n* (%)	10 (4.2)	40 (8.5)	43 (8.0)	0.09
Urinary infection, *n* (%)	11 (4.6)	22 (4.7)	17 (3.2)	0.42
TOAST subtypes, *n* (%)				0.02
Cardioembolism	13 (5.4)	33 (7.0)	25 (4.6)	
Large artery atherosclerosis	133 (55.2)	296 (62.9)	350 (64.9)	
Small artery occlusion	73 (30.3)	122 (25.9)	140 (26.0)	
Other/undetermined	10 (4.2)	7 (1.5)	5 (0.9)	
Undefined	12 (5.0)	13 (2.8)	19 (3.5)	

SD, standard deviation; IQR, interquartile range; NIHSS, National Institute of Health Stroke Scale; HDL, high-density lipoprotein; LDL, low-density lipoprotein; TOAST, trial of org 10172 in acute stroke treatment. ^*∗*^Prediabetes was defined as fasting plasma glucose level of 5.6–6.9 mmol/L or 2-hour oral glucose tolerance test level of 7.8–11.0 mmol/L or haemoglobin A1c level of 5.7–6.4%. ^‡^Newly diagnosed diabetes was defined as fasting plasma glucose ≥ 7.0 mmol/L or 2-hour oral glucose tolerance test ≥ 11.1 mmol/L or haemoglobin A1c ≥ 6.5%.

**Table 2:** Baseline characteristics of abnormal glucose regulation in patients according to different diagnostic criteria.

	Prediabetes^*∗*^				Newly diagnosed diabetes^†^			
	Combined	FPG criteria	OGTT criteria	HbA1c criteria	Combined	FPG criteria	OGTT criteria	HbA1c criteria
Sex (men), *n* (%)	304 (64.8)	172 (61.4)	252 (64.8)	253 (60.1)	314 (58.6)	75 (55.2)	203 (55.3)	229 (63.0)
Age, y, mean ± SD	61.8 ± 12.8	63.5 ± 12.0	63.2 ± 12.5	63.4 ± 12.8	64.2 ± 11.9	60.9 ± 11.8	64.6 ± 11.9	63.4 ± 11.7
NIHSS score at admission, median (IQR)	4 (2–8)	4 (2–8)	4 (2–9)	4 (2–8)	4 (2–8)	5 (2–8)	4 (2–8)	4 (2–7)
Body mass index, kg/m2, mean ± SD	24.7 ± 3.9	25.2 ± 4.0	24.9 ± 3.9	24.8 ± 3.7	25.1 ± 3.8	25.6 ± 4.0	25.3 ± 3.8	25.4 ± 4.0
Triglyceride at admission, mmol/L, mean ± SD	1.7 ± 1.0	1.8 ± 1.2	1.7 ± 0.9	1.7 ± 1.0	1.9 ± 1.2	2.1 ± 1.3	2.0 ± 1.3	2.0 ± 1.3
LDL at admission, mmol/L, mean ± SD	3.1 ± 1.6	3.2 ± 1.2	3.1 ± 1.4	3.2 ± 1.8	3.2 ± 1.5	3.1 ± 1.1	3.2 ± 1.6	3.2 ± 1.2
HDL at admission, mmol/L, mean ± SD	1.2 ± 0.3	1.2 ± 0.5	1.2 ± 0.4	1.2 ± 0.3	1.2 ± 0.4	1.1 ± 0.3	1.2 ± 0.3	1.2 ± 0.4
History of hypertension, *n* (%)	274 (58.2)	181 (64.4)	241 (61.8)	248 (58.6)	339 (62.9)	90 (65.2)	234 (63.2)	223 (61.1)
History of hyperlipidaemia, *n* (%)	68 (14.4)	42 (15.0)	61 (15.6)	57 (13.5)	63 (11.7)	16 (11.6)	39 (10.5)	50 (13.7)
History of atrial fibrillation, *n* (%)	37 (7.9)	21 (7.5)	32 (8.2)	33 (7.8)	25 (4.6)	5 (7.0)	21 (5.7)	16 (4.4)
History of coronary heart disease, *n* (%)	61 (13.0)	51 (18.2)	55 (14.1)	63 (14.9)	70 (13.0)	16 (11.6)	45 (12.2)	53 (14.5)
Smoking, *n* (%)								
Current smoker	160 (34.0)	88 (31.3)	122 (31.3)	137 (32.4)	173 (32.1)	47 (34.1)	118 (31.9)	124 (34.0)
Ever smoker	49 (10.4)	38 (13.5)	48 (12.3)	39 (9.2)	54 (10.0)	13 (9.4)	35 (9.5)	41 (11.2)
Nonsmoker	262 (55.6)	155 (55.2)	220 (56.4)	247 (58.4)	312 (57.9)	78 (56.5)	217 (58.7)	200 (54.8)
Medicine use during hospitalization, *n* (%)								
Oral hypoglycaemic drugs	7 (2.1)	18 (8.4)	16 (5.7)	9 (3.0)	59 (14.3)	24 (20.7)	44 (15.4)	54 (18.8)
Insulin	8 (1.7)	16 (5.7)	12 (3.1)	6 (1.4)	67 (12.4)	41 (29.7)	56 (15.1)	57 (15.6)
Antihypertensive drugs	11 (2.3)	56 (19.9)	30 (7.7)	32 (7.6)	175 (32.5)	70 (50.7)	141 (38.1)	146 (40.0)
Diuretics	13 (2.8)	9 (3.2)	10 (2.6)	8 (1.9)	11 (2.0)	3 (2.2)	8 (2.2)	8 (2.2)
B-blockers	21 (4.5)	14 (5.0)	23 (5.9)	22 (5.2)	21 (3.9)	3 (2.2)	15 (4.1)	13 (3.6)
Statin	253 (53.7)	145 (51.6)	216 (55.4)	232 (54.9)	277 (51.4)	69 (50.0)	193 (52.2)	196 (53.7)
Antiplatelet	304 (64.5)	179 (63.7)	255 (65.4)	264 (62.4)	332 (61.6)	79 (57.3)	236 (63.8)	229 (62.7)
Anticoagulation	27 (5.7)	23 (8.2)	32 (8.2)	22 (5.2)	36 (6.7)	3 (2.2)	20 (5.4)	25 (6.9)
Pulmonary infection, *n* (%)	40 (8.5)	29 (10.3)	33 (8.5)	33 (7.8)	43 (8.0)	9 (6.5)	32 (8.7)	19 (5.2)
Urinary infection, *n* (%)	22 (4.7)	7 (2.5)	17 (4.4)	22 (5.2)	17 (3.2)	6 (4.4)	11 (3.0)	9 (2.5)
TOAST subtypes, *n* (%)								
Cardioembolism	33 (7.0)	16 (5.7)	29 (7.4)	31 (7.3)	25 (4.6)	2 (1.5)	19 (5.1)	14 (3.8)
Large artery atherosclerosis	296 (62.9)	185 (65.8)	238 (61.0)	259 (61.2)	350 (64.9)	96 (69.6)	244 (66.0)	230 (63.0)
Small artery occlusion	122 (25.9)	72 (25.6)	106 (27.2)	114 (27.0)	140 (26.0)	34 (24.6)	96 (26.0)	101 (27.7)
Other/undetermined	7 (1.5)	1 (0.4)	5 (1.3)	6 (1.4)	5 (0.9)	3 (2.2)	1 (0.3)	5 (1.4)
Undefined	13 (2.8)	7 (2.5)	12 (3.1)	13 (3.1)	19 (3.5)	3 (2.2)	10 (2.7)	15 (4.1)

SD, standard deviation; IQR, interquartile range; NIHSS, National Institute of Health Stroke Scale; HDL, high-density lipoprotein; LDL, low-density lipoprotein. ^*∗*^Prediabetes: FPG criteria was defined as fasting plasma glucose level of 5.6–6.9 mmol/L, OGTT criteria was defined as 2-hour oral glucose tolerance test level of 7.8–11.0 mmol/L; HbA1c criteria was defined as haemoglobin A1c level of 5.7–6.4%; and combined criteria was defined as using FPG, OGTT, or HbA1c criteria combined. ^†^Newly diagnosed diabetes: FPG criteria was defined as fasting plasma glucose ≥ 7.0 mmol/L; OGTT criteria was defined as 2-hour oral glucose tolerance test ≥ 11.1 mmol/L; HbA1c criteria was defined as haemoglobin A1c ≥ 6.5%; and combined criteria was defined as FPG, OGTT, or HbA1c criteria combined.
